# 4,4′-{[1,2-Phenyl­enebis(methyl­ene)]bis­(­oxy)}di­benzoic acid di­methyl­formamide disolvate

**DOI:** 10.1107/S1600536814006795

**Published:** 2014-04-12

**Authors:** Zhen-Zhe Qiu, Bi Jing, Qiu-Xia Li, Ai-Xin Zhu

**Affiliations:** a Faculty of Chemistry and Chemical Engineering, Yunnan Normal University, Kunming 650050, People’s Republic of China

## Abstract

In the title solvate, C_22_H_18_O_6_·2C_3_H_7_NO, the complete dicarboxylic acid molecule is generated by a crystallographic twofold axis, which bisects the central benzene ring and one *N*,*N*-di­methyl­formamide solvent mol­ecule. The dihedral angle between the central and pendant benzene rings is 54.53 (5)° while that between the pendant rings is 45.44 (5)°. In the crystal, the acid molecules are linked to the solvent molecules *via* O—H⋯O and weak C—H⋯O hydrogen bonds. Further weak C—H⋯O inter­actions link adjacent acid mol­ecules into a three-dimensional network.

## Related literature   

For multi­carb­oxy­lic acid ligands and derivatives used in the synthesis of porous metal-organic frameworks, see: Eddaoudi *et al.* (2002 ▶); Eubank *et al.* (2011 ▶); Zhang *et al.* (2012 ▶). For structures constructed by the acid mol­ecule of the title compound, see: Cao *et al.* (2009*a* ▶); Hu *et al.* (2013 ▶). For [Zn(1,2-BAB)(4,4′-bipy)_1/2_]_*n*_ (H_2_BAB =4,4′-{[1,2-phenylenebis(methylene)]bis(oxy)}dibenzoic acid), see Cao *et al.* (2009*a* ▶) and for [Cd(1,2-BAB)_2_(phen)_2_]_*n*_, see: Cao *et al.* (2009*b* ▶). For the synthesis of the title compound, see: Cao *et al.* (2009*a* ▶); Rajakumar *et al.* (2009 ▶).
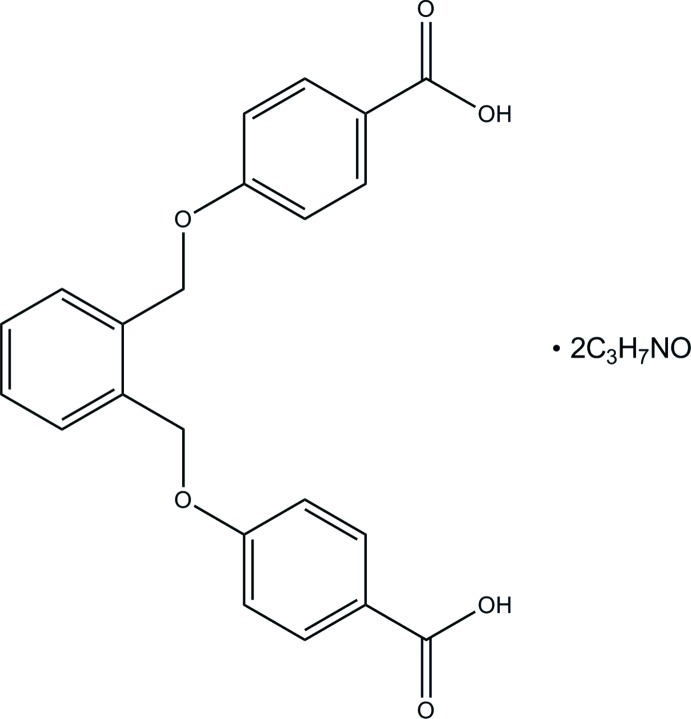


## Experimental   

### 

#### Crystal data  

C_22_H_18_O_6_·2C_3_H_7_NO
*M* *_r_* = 524.56
Monoclinic, 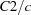

*a* = 12.568 (3) Å
*b* = 11.081 (2) Å
*c* = 19.688 (4) Å
β = 98.04 (3)°
*V* = 2715.0 (9) Å^3^
*Z* = 4
Mo *K*α radiation
μ = 0.09 mm^−1^
*T* = 293 K
0.37 × 0.26 × 0.21 mm

#### Data collection  

Rigaku R-AXIS RAPID IP diffractometer
Absorption correction: multi-scan (*ABSCOR*; Higashi, 1995 ▶) *T*_min_ = 0.783, *T*_max_ = 1.000
12992 measured reflections
3112 independent reflections
2522 reflections with *I* > 2σ(*I*)
*R*_int_ = 0.023

#### Refinement  

*R*[*F* ^2^ > 2σ(*F* ^2^)] = 0.042
*wR*(*F* ^2^) = 0.132
*S* = 1.08
3112 reflections
179 parameters
H atoms treated by a mixture of independent and constrained refinement
Δρ_max_ = 0.20 e Å^−3^
Δρ_min_ = −0.18 e Å^−3^

### 

Data collection: *RAPID-AUTO* (Rigaku, 1998 ▶); cell refinement: *RAPID-AUTO*; data reduction: *CrystalClear* (Rigaku/MSC, 2002 ▶); program(s) used to solve structure: *SHELXS97* (Sheldrick, 2008 ▶); program(s) used to refine structure: *SHELXL97* (Sheldrick, 2008 ▶); molecular graphics: *DIAMOND* (Brandenburg, 1999 ▶); software used to prepare material for publication: *SHELXTL* (Sheldrick, 2008 ▶).

## Supplementary Material

Crystal structure: contains datablock(s) I, global. DOI: 10.1107/S1600536814006795/bg2524sup1.cif

Structure factors: contains datablock(s) I. DOI: 10.1107/S1600536814006795/bg2524Isup2.hkl

Supporting information file. DOI: 10.1107/S1600536814006795/bg2524Isup3.cdx

Supporting information file. DOI: 10.1107/S1600536814006795/bg2524Isup4.cml

CCDC reference: 994050

Additional supporting information:  crystallographic information; 3D view; checkCIF report

## Figures and Tables

**Table 1:** Hydrogen-bond geometry (Å, °)

*D*—H⋯*A*	*D*—H	H⋯*A*	*D*⋯*A*	*D*—H⋯*A*
O1—H1*A*⋯O4	0.90 (2)	1.71 (2)	2.6064 (14)	174 (2)
C3—H3*A*⋯O1^i^	0.93	2.55	3.3714 (17)	147
C8—H8*B*⋯O2^ii^	0.97	2.58	3.4920 (18)	157
C14—H14*A*⋯O2	0.93	2.50	3.2110 (19)	134

Symmetry codes: (i) 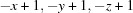; (ii) 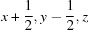.

**Fig. 1.**
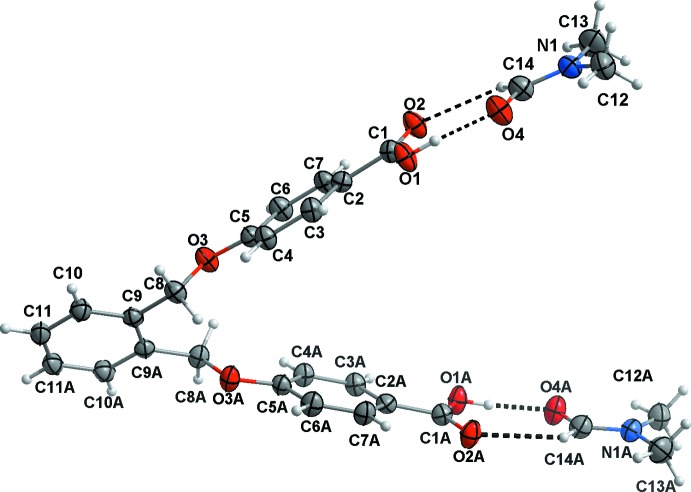
ORTEP view of the title compound drawn with 30% probability displacement ellipsoids for the non-H atoms. The intermolecular interactions between the acid and solvent molecules are shown as dashed lines. Symmetry code (A): 1-x, y, -z+1/2.

**Fig. 2.**
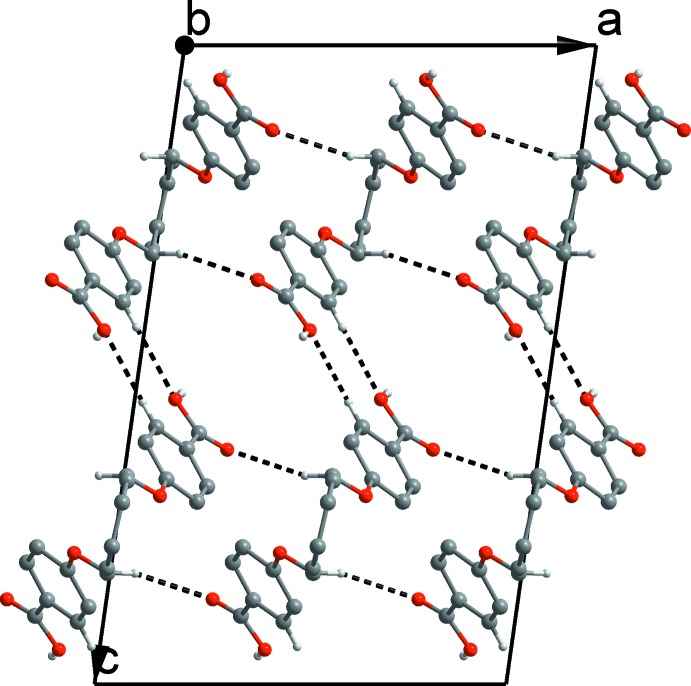
Packing of the acid molecules in the title compound viewed along the b-axis showing the hydrogen bonding interactions with dashed lines. H atoms not involved in H-bonding have been omitted for clarity.

## supplementary crystallographic information

### 1. Comment

In the past few years, multicarboxylic acids and their derivatives have attracted increasing attention as an important class of ligands used for the synthesis of porous metal organic framework compounds (Eddaoudi *et al.* 2002; Eubank *et al.* 2011; Zhang *et al.* 2012). The acid molecule in the title compound, as a conformationally flexible V-shaped long bicarboxylate ligand, has been already used to synthesize entangled frameworks having both polyrotaxane and polycatenane characteristics that also achieve different topological structures in the entangled system (Cao *et al.* 2009*a*; Hu *et al.* 2013). Although there are crystal structure reports in the literature regarding the title multicarboxylic acid, no crystallographic study has been already performed on the ligand itself.

The crystal structure of the title compound is composed of 4,4'-(1,2-phenylenebis(methylene))bis(oxy)dibenzoic (dicarboxylic) acid and *N*,*N*-dimethylformamide and has monoclinic symmetry (space group: *C*2/*c*). The acid molecule adopts an *E* configuration, and contains a crystallographic *C*2 axis passing through the central benzene group (Fig. 1). The dihedral angles between the benzene rings are 45.44 (5)° and 54.53 (5)°, values which are significantly smaller than those in metal organic frameworks containing the acid molecules with an E configuration (where the acid molecule loses both protons from the carboxylic groups); for example, [Zn(1,2-BAB)(4,4'-bipy)_1/2_]_n_ (4,4'-bipy = 4,4'-Bipyridine) and [Cd(1,2-BAB)_2_(phen)_2_]_n_ (phen = 1,10-phenanthroline) the dihedral angles range from 58.2 (1)° to 70.9 (1)° and from 65.6 (1)° to 84.7 (1)°, respectively (Cao *et al.* 2009*a*,*b*).

In the crystal, the acid molecule are linked to the solvent molecules by a strong O—HO and a weak C—HO hydrogen bond [Table 1 (entries 1 and 4) and Fig. 1]. Besides, weak intermolecular C—H···O interactions link the adjacent acid molecules into a three-dimensional network [Table 1 (entries 2 and 3) and Fig 2].

### 2. Experimental

The ligand 4,4'-(1,2-phenylenebis(methylene))bis(oxy)dibenzoic acid was synthesized according to the literature method (Cao *et al.* 2009*a*; Rajakumar *et al.* 2009). A mixture of 4,4'-(1,2-phenylenebis(methylene))bis(oxy)dibenzoic acid (37.8 mg, 0.1 mmol) and DMF (4 ml) was placed in a Teflon-lined stainless steel vessel (15 ml) and heated at 368 k for 48 h and then cooled to room temperature at a rate of 5 K h-1. The resulting colorless solution slowly evaporated in air for over two weeks and colorless block crystals of the title compound suitable for X-ray diffraction were obtained.

### 3. Refinement

The positions of the hydroxyl hydrogen H1A could be obtained from the difference electron-density map, and the other H atoms were placed in idealized positions (O—H = 0.82 Å and C—H = 0.93–0.97 Å) and refined as riding atoms with *U*_iso_(H) = 1.2*U*_eq_(C, N) and *U*_iso_(H) = 1.5*U*_eq_(O, C_methyl_).

### Crystal data

**Table:**

C_22_H_18_O_6_·2C_3_H_7_NO	*Z* = 4
*M**_r_* = 524.56	*F*(000) = 1112
Monoclinic, *C*2/*c*	*D*_x_ = 1.283 Mg m^−^^3^
Hall symbol: -C 2yc	Mo *K*α radiation, λ = 0.71073 Å
*a* = 12.568 (3) Å	µ = 0.09 mm^−^^1^
*b* = 11.081 (2) Å	*T* = 293 K
*c* = 19.688 (4) Å	Block, colorless
β = 98.04 (3)°	0.37 × 0.26 × 0.21 mm
*V* = 2715.0 (9) Å^3^	

### Data collection

**Table:**

Rigaku R-AXIS RAPID IP diffractometer	3112 independent reflections
Radiation source: fine-focus sealed tube	2522 reflections with *I* > 2σ(*I*)
Graphite monochromator	*R*_int_ = 0.023
ω scans	θ_max_ = 27.5°, θ_min_ = 3.1°
Absorption correction: multi-scan (*ABSCOR*; Higashi, 1995)	*h* = −16→16
*T*_min_ = 0.783, *T*_max_ = 1.000	*k* = −14→14
12992 measured reflections	*l* = −25→24

### Refinement

**Table:**

Refinement on *F*^2^	Secondary atom site location: difference Fourier map
Least-squares matrix: full	Hydrogen site location: inferred from neighbouring sites
*R*[*F*^2^ > 2σ(*F*^2^)] = 0.042	H atoms treated by a mixture of independent and constrained refinement
*wR*(*F*^2^) = 0.132	*w* = 1/[σ^2^(*F*_o_^2^) + (0.0749*P*)^2^ + 0.5159*P*] where *P* = (*F*_o_^2^ + 2*F*_c_^2^)/3
*S* = 1.08	(Δ/σ)_max_ = 0.004
3112 reflections	Δρ_max_ = 0.20 e Å^−^^3^
179 parameters	Δρ_min_ = −0.18 e Å^−^^3^
0 restraints	Extinction correction: *SHELXL97* (Sheldrick, 2008), Fc^*^=kFc[1+0.001xFc^2^λ^3^/sin(2θ)]^-1/4^
Primary atom site location: structure-invariant direct methods	Extinction coefficient: 0.0067 (9)

### Special details

**Table:**

Geometry. All e.s.d.'s (except the e.s.d. in the dihedral angle between two l.s. planes) are estimated using the full covariance matrix. The cell e.s.d.'s are taken into account individually in the estimation of e.s.d.'s in distances, angles and torsion angles; correlations between e.s.d.'s in cell parameters are only used when they are defined by crystal symmetry. An approximate (isotropic) treatment of cell e.s.d.'s is used for estimating e.s.d.'s involving l.s. planes.
Refinement. Refinement of *F*^2^ against ALL reflections. The weighted *R*-factor *wR* and goodness of fit *S* are based on *F*^2^, conventional *R*-factors *R* are based on *F*, with *F* set to zero for negative *F*^2^. The threshold expression of *F*^2^ > σ(*F*^2^) is used only for calculating *R*-factors(gt) *etc*. and is not relevant to the choice of reflections for refinement. *R*-factors based on *F*^2^ are statistically about twice as large as those based on *F*, and *R*- factors based on ALL data will be even larger.

### Fractional atomic coordinates and isotropic or equivalent isotropic displacement parameters (Å^2^)

**Table:**

	*x*	*y*	*z*	*U*_iso_*/*U*_eq_	
O1	0.40156 (8)	0.62429 (9)	0.44602 (5)	0.0603 (3)	
H1A	0.3863 (16)	0.701 (2)	0.4567 (11)	0.097 (6)*	
O2	0.25800 (8)	0.64860 (9)	0.36718 (5)	0.0634 (3)	
O3	0.40553 (7)	0.12257 (8)	0.29461 (5)	0.0542 (3)	
O4	0.37129 (9)	0.84725 (9)	0.48090 (5)	0.0697 (3)	
N1	0.27647 (9)	1.01724 (11)	0.45297 (6)	0.0612 (3)	
C1	0.33217 (9)	0.58739 (11)	0.39294 (6)	0.0454 (3)	
C2	0.35606 (9)	0.46508 (10)	0.36834 (6)	0.0426 (3)	
C3	0.43792 (10)	0.39435 (11)	0.40211 (6)	0.0466 (3)	
H3A	0.4797	0.4243	0.4412	0.056*	
C4	0.45868 (10)	0.27970 (11)	0.37878 (6)	0.0477 (3)	
H4A	0.5140	0.2333	0.4017	0.057*	
C5	0.39532 (9)	0.23540 (10)	0.32053 (6)	0.0442 (3)	
C6	0.31335 (10)	0.30568 (12)	0.28570 (7)	0.0523 (3)	
H6A	0.2716	0.2758	0.2466	0.063*	
C7	0.29425 (10)	0.41968 (12)	0.30942 (6)	0.0497 (3)	
H7A	0.2398	0.4667	0.2860	0.060*	
C8	0.49311 (10)	0.04951 (11)	0.32584 (6)	0.0477 (3)	
H8A	0.4838	0.0308	0.3728	0.057*	
H8B	0.5604	0.0927	0.3264	0.057*	
C9	0.49515 (9)	−0.06479 (10)	0.28507 (6)	0.0431 (3)	
C10	0.49017 (10)	−0.17419 (11)	0.31849 (7)	0.0522 (3)	
H10A	0.4834	−0.1747	0.3649	0.063*	
C11	0.49509 (11)	−0.28268 (11)	0.28426 (7)	0.0574 (3)	
H11A	0.4917	−0.3552	0.3076	0.069*	
C12	0.33915 (15)	1.08831 (15)	0.50625 (9)	0.0759 (5)	
H12A	0.4015	1.0434	0.5255	0.114*	
H12B	0.2962	1.1064	0.5416	0.114*	
H12C	0.3613	1.1622	0.4869	0.114*	
C13	0.18927 (15)	1.07654 (18)	0.40971 (12)	0.0903 (6)	
H13A	0.1600	1.0227	0.3737	0.135*	
H13B	0.2158	1.1479	0.3901	0.135*	
H13C	0.1342	1.0983	0.4367	0.135*	
C14	0.29748 (12)	0.90176 (14)	0.44604 (8)	0.0617 (4)	
H14A	0.2537	0.8585	0.4126	0.074*	

### Atomic displacement parameters (Å^2^)

**Table:**

	*U*^11^	*U*^22^	*U*^33^	*U*^12^	*U*^13^	*U*^23^
O1	0.0695 (6)	0.0499 (5)	0.0549 (5)	0.0117 (4)	−0.0139 (4)	−0.0105 (4)
O2	0.0590 (6)	0.0563 (5)	0.0690 (6)	0.0145 (4)	−0.0118 (5)	−0.0082 (4)
O3	0.0496 (5)	0.0479 (5)	0.0597 (5)	0.0077 (4)	−0.0111 (4)	−0.0126 (4)
O4	0.0791 (7)	0.0580 (6)	0.0679 (6)	0.0169 (5)	−0.0038 (5)	−0.0100 (5)
N1	0.0563 (7)	0.0543 (6)	0.0735 (7)	0.0069 (5)	0.0113 (6)	0.0046 (5)
C1	0.0462 (6)	0.0455 (6)	0.0433 (6)	0.0008 (5)	0.0020 (5)	0.0015 (5)
C2	0.0419 (6)	0.0432 (5)	0.0417 (5)	−0.0010 (4)	0.0029 (4)	0.0006 (4)
C3	0.0496 (6)	0.0462 (6)	0.0405 (5)	−0.0010 (5)	−0.0055 (5)	−0.0021 (5)
C4	0.0472 (6)	0.0463 (6)	0.0457 (6)	0.0047 (5)	−0.0069 (5)	0.0002 (5)
C5	0.0422 (6)	0.0433 (6)	0.0456 (6)	−0.0002 (4)	0.0012 (5)	−0.0034 (5)
C6	0.0469 (6)	0.0544 (7)	0.0505 (6)	0.0023 (5)	−0.0108 (5)	−0.0082 (5)
C7	0.0440 (6)	0.0508 (6)	0.0504 (6)	0.0060 (5)	−0.0077 (5)	−0.0013 (5)
C8	0.0475 (6)	0.0476 (6)	0.0457 (6)	0.0048 (5)	−0.0016 (5)	−0.0023 (5)
C9	0.0368 (5)	0.0436 (6)	0.0477 (6)	0.0012 (4)	0.0015 (4)	−0.0005 (4)
C10	0.0530 (7)	0.0503 (6)	0.0535 (6)	0.0002 (5)	0.0078 (5)	0.0066 (5)
C11	0.0549 (7)	0.0422 (6)	0.0753 (8)	−0.0006 (5)	0.0102 (6)	0.0089 (6)
C12	0.0867 (11)	0.0567 (8)	0.0852 (11)	−0.0003 (8)	0.0155 (9)	−0.0087 (8)
C13	0.0650 (10)	0.0815 (11)	0.1215 (16)	0.0154 (9)	0.0034 (10)	0.0215 (11)
C14	0.0602 (8)	0.0604 (8)	0.0635 (8)	0.0056 (6)	0.0056 (6)	−0.0060 (6)

### Geometric parameters (Å, º)

**Table:**

O1—C1	1.3285 (15)	C6—H6A	0.9300
O1—H1A	0.90 (2)	C7—H7A	0.9300
O2—C1	1.2059 (14)	C8—C9	1.5016 (16)
O3—C5	1.3633 (14)	C8—H8A	0.9700
O3—C8	1.4338 (14)	C8—H8B	0.9700
O4—C14	1.2323 (17)	C9—C10	1.3848 (16)
N1—C14	1.3175 (19)	C9—C9^i^	1.403 (2)
N1—C13	1.448 (2)	C10—C11	1.3837 (18)
N1—C12	1.453 (2)	C10—H10A	0.9300
C1—C2	1.4841 (16)	C11—C11^i^	1.372 (3)
C2—C3	1.3868 (16)	C11—H11A	0.9300
C2—C7	1.3966 (16)	C12—H12A	0.9600
C3—C4	1.3879 (17)	C12—H12B	0.9600
C3—H3A	0.9300	C12—H12C	0.9600
C4—C5	1.3911 (16)	C13—H13A	0.9600
C4—H4A	0.9300	C13—H13B	0.9600
C5—C6	1.3929 (16)	C13—H13C	0.9600
C6—C7	1.3794 (18)	C14—H14A	0.9300
			
C1—O1—H1A	109.6 (13)	C9—C8—H8A	110.0
C5—O3—C8	117.65 (9)	O3—C8—H8B	110.0
C14—N1—C13	121.73 (14)	C9—C8—H8B	110.0
C14—N1—C12	120.34 (13)	H8A—C8—H8B	108.4
C13—N1—C12	117.91 (14)	C10—C9—C9^i^	118.91 (7)
O2—C1—O1	122.79 (11)	C10—C9—C8	118.63 (11)
O2—C1—C2	123.80 (11)	C9^i^—C9—C8	122.44 (7)
O1—C1—C2	113.41 (10)	C11—C10—C9	121.41 (12)
C3—C2—C7	118.94 (11)	C11—C10—H10A	119.3
C3—C2—C1	122.01 (10)	C9—C10—H10A	119.3
C7—C2—C1	119.05 (10)	C11^i^—C11—C10	119.68 (8)
C4—C3—C2	121.32 (10)	C11^i^—C11—H11A	120.2
C4—C3—H3A	119.3	C10—C11—H11A	120.2
C2—C3—H3A	119.3	N1—C12—H12A	109.5
C3—C4—C5	118.90 (10)	N1—C12—H12B	109.5
C3—C4—H4A	120.5	H12A—C12—H12B	109.5
C5—C4—H4A	120.5	N1—C12—H12C	109.5
O3—C5—C4	124.01 (10)	H12A—C12—H12C	109.5
O3—C5—C6	115.48 (10)	H12B—C12—H12C	109.5
C4—C5—C6	120.49 (11)	N1—C13—H13A	109.5
C7—C6—C5	119.81 (10)	N1—C13—H13B	109.5
C7—C6—H6A	120.1	H13A—C13—H13B	109.5
C5—C6—H6A	120.1	N1—C13—H13C	109.5
C6—C7—C2	120.54 (10)	H13A—C13—H13C	109.5
C6—C7—H7A	119.7	H13B—C13—H13C	109.5
C2—C7—H7A	119.7	O4—C14—N1	124.37 (14)
O3—C8—C9	108.45 (9)	O4—C14—H14A	117.8
O3—C8—H8A	110.0	N1—C14—H14A	117.8

Symmetry code: (i) −*x*+1, *y*, −*z*+1/2.

### Hydrogen-bond geometry (Å, º)

**Table:**

*D*—H···*A*	*D*—H	H···*A*	*D*···*A*	*D*—H···*A*
O1—H1*A*···O4	0.90 (2)	1.71 (2)	2.6064 (14)	174 (2)
C3—H3*A*···O1^ii^	0.93	2.55	3.3714 (17)	147
C8—H8*B*···O2^iii^	0.97	2.58	3.4920 (18)	157
C14—H14*A*···O2	0.93	2.50	3.2110 (19)	134

Symmetry codes: (ii) −*x*+1, −*y*+1, −*z*+1; (iii) *x*+1/2, *y*−1/2, *z*.
